# Evaluation of the Effect of Water on Three Different Light Cured Composite Restorative Materials Stored in Water: An In Vitro Study

**DOI:** 10.1155/2012/640942

**Published:** 2012-01-22

**Authors:** Basawaraj Biradar, Sudharani Biradar, Arvind MS

**Affiliations:** ^1^Department of Conservative Dentistry & Endodontics, Rural Dental College, Pravara Medical Trust Campus, Loni, Rahata Taluka, Ahmednagar District, India; ^2^Department of Oral Medicine, Diagnosis and Radiology, Rural Dental College, PMT Loni, Rahata Taluka, Ahemadnagar Dist 413736, India; ^3^Department of Conservative Dentistry and Endodontics, Sri Rajiv Gandhi College of Dental Sciences, Banglore 560032, India

## Abstract

*Objectives*. The objective of this in vitro study was to investigate whether weight gain or loss in the three different composites occurs due to water absorption when they are stored in water. *Methods*. The composite restorative materials selected for this study included a microfine hybrid (Synergy) and two nanofilled composite restorative materials (Ceram X and Filtek Supreme Ultra). Twenty specimens of each material were fabricated of each composite material. Group A: Filtek Supreme Ultra, Group B: Synergy, Group C: Ceram X. Then all the specimens were stored in 10 ml Distilled water containing test tubes and placed in incubator at 37°C for six weeks. The weight changes of these specimens were measured daily for the first week and later once a week for next five weeks by using an electrical analytical balance. *Results.* The data was analyzed by one-way analysis of variance and Student's *t* test. All groups showed maximum amount of water absorption in the first week than gradual decrease in the water absorption from the second to the sixth week, as compared to the first week and there is no statistically significant difference between the groups tested. *Conclusion*. All the composite restorative material absorbs some amount of water. The water absorption of the composite may decrease the physical and mechanical properties of the composites; hence it is necessary to consider the type of the material before starting the treatment.

## 1. Introduction

 The attractiveness of tooth-colored restorations has stimulated research in this particular area of operative dentistry during recent years; patients are increasingly demanding esthetic restorations not only in the anterior teeth but also in the posterior teeth. Dental material composites are today used widely, not only because of their esthetic properties but also for the ability to adhere to tooth substance [1, 2].

Mechanical properties of composites are not only influenced by their chemical composition but also by the environment to which they are exposed. The corrosion process promoted by water and the presence of constant load on the surface of resin are responsible for the appearance and propagation of interfacial debonding, matrix cracking, superficial flaws, filler dissolution, and filler particle dislodgement [3]. 

 Nanotechnology is also known as molecular nanotechnology, or molecular engineering is the production of functional material and the structures in the range of 0.1 to 100 nanometers by various physical and chemical methods. The intense interest in nanomaterial, to provide dramatic improvement in electrical, chemical, mechanical, and optical properties [4].

 The problem associated with these restorative materials is water absorption as they are continuously bathed in saliva; for resin-based composite materials water absorption may induce weakening of the matrix and breakdown of resin filler interface. It is also expected that absorption of water will be accompanied by hygroscopic expansion of composite which may be able to compensate for the effect of polymerization shrinkage and relieve stresses [1].

 The dimensional changes in composite restorative materials placed in the cavity are the result of shrinkage of resin monomer during polymerization. Shrinkage is compensated by the expansion resulting from the water absorption of set resin. This fact has drawn much attention regarding the adaptation of composite to the dental cavity walls [2, 5, 6]. 

Water sorption actually increases with cross-linker concentration, suggesting that the chemical nature of cross-linking agent may supercede the effect of higher molecular density; high level of porosity or microvoids has also been shown to facilitate fluid transport into and out of the polymer.

So the aim of this study is to evaluate the effect of water on three different light-cured composite restorative materials stored in water. 

## 2. Materials and Methods

Twenty specimens from each composite material (Table 1) were prepared using brass mold (6 mm diameter × 2 mm height). The composite material was covered with acetate strips and compressed between 2 glass slabs to remove voids and extrude excess composite material. The composite was then light cured through the acetate strip for 40 seconds on both the sides by using QTH light-curing unit (QHL-75, Dentsply). The light-curing unit was held as close to the specimen as possible and cured at an intensity of 450 mW/cm^2^. The tip diameter of the light-curing unit was 11 mm in diameter [1].

**Figure 1:**
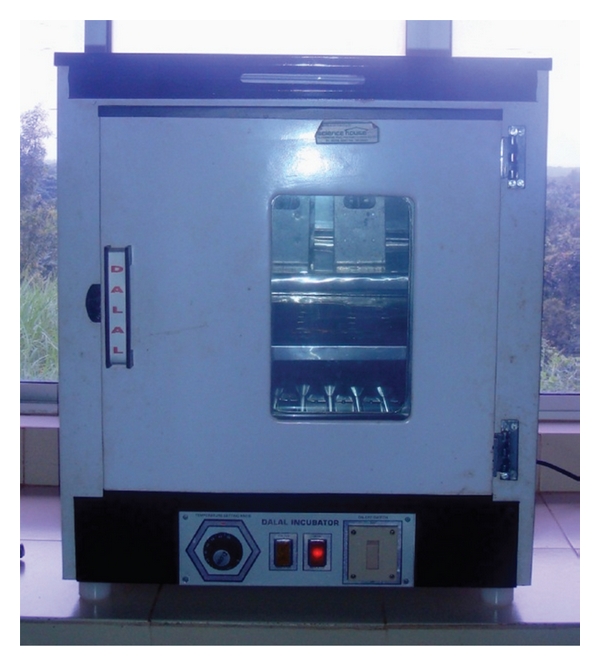
Specimens incubated for 37°C for six weeks.

 Following light curing, the specimens were removed from the mold and finished with carborundum paper and later polished with coarse, medium, and fine Sof-Lex discs (3M ESPE) in respective orders. The specimens were then weighed by electrical analytical balance (DANVER INSTRUMENT), and each specimen was placed in separate test tube (BOROSIL) containing 10 mL distilled water. The specimens were sealed in a test tube with cotton pellet and placed in an incubator for 6 weeks at 37°C (Figure 1).

 Weight change of the specimen was measured according to the ISO 4049 (International Organization of Standardization) original plan (1985), and water solubility of the specimen was determined as per ADA specification no. 8 (1978) [7].

**Figure 2:**
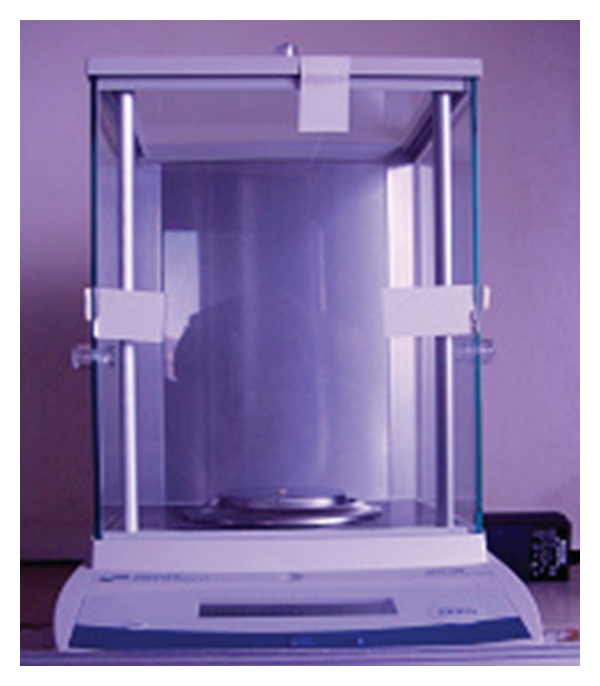
Specimen placed in electrical analytical balance.

 After 24 hours, the specimens were removed and placed on the filter paper (Whatman) for a period of 1 min to drain excess water and then weighed accurately using an electrical analytical balance (Figure 2).

After weighing the specimens, they were transferred to test tubes filled with 10 mL of fresh distilled water. 

The procedure was repeated every day for the first week and then once a week for the next five weeks. 

Data obtained was analyzed statistically using analysis of variance (ANOVA) and Student's *t* test.

The data was analyzed using multivariate approach of repeated measures analysis of variance (ANOVA) of SPSS Version 13.00. 

## 3. Results

The data was analyzed by One-way analysis of variance and Student's *t* test.

All groups showed maximum amount of water absorption in the first week than gradual decrease in the water absorption from the second to the sixth week (Tables 2 and 3).

There was no significant difference noted among the materials (*P* > 0.05). As a result, the difference between the groups was not compared. 

**Figure 3:**
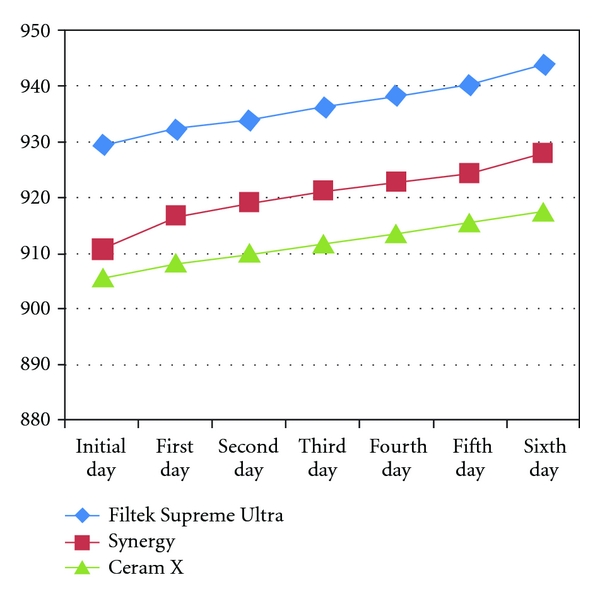
Line graph showing changes in the weight ofall study groups measured daily (*x-*axis measures the days and *y-*axis measures the weight in grams).

**Figure 4:**
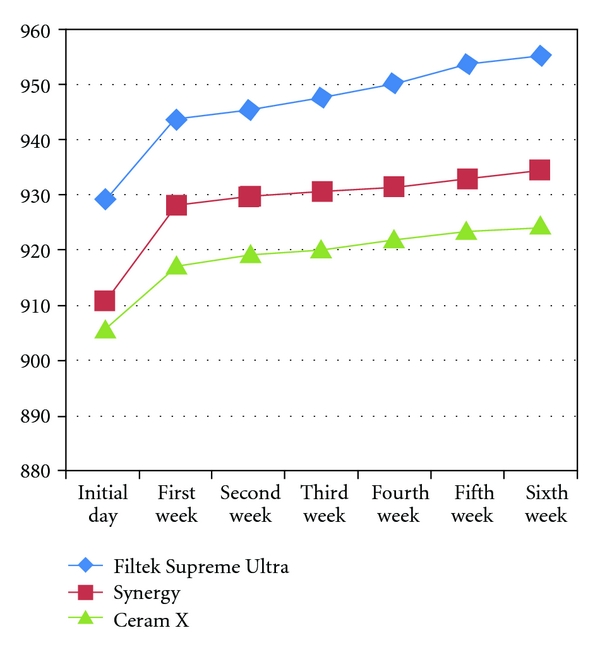
Line graph showing changes in the weight all study groups measured weekly (*x-*axis measures the weeks and *y*-axis measures the weight in grams).

Figure 3 shows the weight change of all the specimens of one week water storage measured daily, while Figure 4 shows the weight changes of all specimens during the test period measured weekly. 

## 4. Discussion

Weight change in water was evaluated because saliva is a dilute fluid consisting of 99.5% of water. The concentrations of dissolved solids (organic or inorganic) are characterized by wide variations, both between individual and within a single individual. Due to this variation, water was used as test standard [1].

 Brass was chosen for this study, because many of its physical properties are similar to those of the tooth substance. For example, Young's modulus of brass is very close to that of enamel while its hardness lies in between the hardness of enamel and dentin. The coefficient of thermal expansion of brass is similar to that the tooth structure [2, 8].

Quartz-tungsten-halogen light-curing unit was used having an intensity of 450 mW/ cm^2^ and wavelength between 400 and 500 nm which was sufficient to cure composite specimens up to a depth of 2 mm [9, 10]. 

Acetate strips were used to prevent the formation of oxygen-inhibited layer on the surface of the composite [11].

The factors which affect the amount of water absorption of the composite restoration materials are the resin content, filler content, curing time, distance from composite cured and the coupling agent [12–16]. The more the filler content of the composite the lesser will be the water absorption [12, 17]. The proper the bonding of the coupling agent the lesser the water absorption [1, 18]. 

 This study showed maximum amount of water absorption in the first week of the experiment [1, 19–21]. The dimensional changes in composite restorative materials in the first week were the result of shrinkage of resin monomer during polymerization in the first week [22]. Shrinkage is compensated by the expansion resulting from the water absorption of set resin. This fact has drawn much attention regarding the adaptation of composite to the dental cavity walls [2, 4, 5].

The study done by Knobloch et al. also showed maximum amount of water absorption in the first week of the experiment [20]. The study done by keyf and Yalçin also showed maximum amount of water absorption in the first week of the experiment [1]. The study done by Hegde and Biradar also showed maximum amount of water absorption in the first week of the experiment [19].

There is no statistically significant difference between the groups tested, but this study showed Synergy absorbs maximum amount of water compared to Filtek Supreme Ultra and Ceram X in the first week of the study (Table 4). This is because Synergy contains increased resin to filler ratio, it showed maximum amount of water absorption [1, 23]. However, in this study only the relationship among immersion time, the water absorption of the resin, and the thickness of the specimen is focused. Weight loss due to dissolution was not included in the measurement; the diffusion coefficient and thickness of the specimen were affected by the amount of water absorption [2]. 

In this study all material showed >90% of final volumetric expansion and change in weight within 7 days thereafter followed slower and more gradual increase in volume and weight [7]. In this study Filtek Supreme Ultra showed maximum amount of water absorption from the second to the sixth week compared to Ceram X and Synergy (Table 5). This two-stage expansion may be caused due to hydrolytic degradation of monomer bonds or stretching of these bonds beyond their elastic limit causing them to rupture [24].

 The study done by Iwami et al. also showed more than 90% of the water absorption occurred in the first week [25]. 

The increase in the dimension shown by the materials may be beneficial in relieving some of internal polymerization shrinkage stresses and increase the longevity of the adhesive union to surrounding tooth [20].

Studies on the amount of weight loss due to dissolution, diffusion coefficient, thickness of the specimen, and changes in physical and mechanical properties are further required before conclusive clinical assessment.

## 5. Conclusion

The present in vitro study evaluated the effect of water on microfine hybrid (Synergy) and two different nanofilled (Filtek Supreme Ultra and Ceram X) composite restorative materials.

The following conclusions were drawn.

1. All the groups showed some amount weight gain due to water absorption.
2. All groups showed maximum amount of weight gain in the first week and slowly decrease in the amount of water absorption from second to sixth week.
3. There is no statistically significant difference between the groups tested.

## Figures and Tables

**Table 1:** Materials used.

Materials used	Manufacturer	Composite type	Matrix
Filtek Supreme Ultra (Group A)	3M ESPE	Nanocomposite	Nanocomposite, Universal restorative material. Aggregated zirconia/silica cluster filler, with an average particle size of 0.6–1.4 micron
Synergy (Group B)	Coltene Whaledent	Nanohybrid	Microfine hybrid BisGMA, BisEMA, TEGDMA, strontium glass, barium glass, Amorphous silica
Ceram X (Group C)	Dentsply	Nanocomposite	Nanoceramic methacrylate-modified polysiloxane, dimethacrylate resin, fluorescence pigment, camphorquinone.

**Table 2:** The mean weights of three composite specimen measured daily during the first week of the observation.

	No. of observations	Group A	Group B	Group C
Initial	20	929.2 (28.140)	910. 7 (18.979)	905. 6 (15.806)
First day	20	932.5 (26.963)	916. 7 (19.074)	908. 3 (15.267)
Second day	20	933.8 (27.976)	919. 1 (16.368)	910. 0 (15.499)
Third day	20	936.2 (26.246)	921. 2 (15.161)	911. 7 (15.291)
Fourth day	20	938.0 (23.576)	922. 8 (15.087)	913. 5 (14.855)
Fifth day	20	940. 4 (25.124)	924. 4 (15.916)	915. 5 (15.157)
Sixth day	20	943. 8 (25.614)	928. 0 (14.706)	917. 5 (14.652)

(standard deviations are given within brackets).

**Table 3:** The mean weights of three composite specimen measured daily during the entire period of the observation.

	No. of observations	Group A	Group B	Group C
Initial	20	929.2 (28.140)	910. 7 (18.979)	905. 6 (15.806)
First week	20	943. 8 (25.614)	928. 0 (14.706)	917. 5 (14.652)
Second week	20	945. 4 (25.488)	929. 5 (14.580)	919. 4 (13.808)
Third week	20	947. 7 (26.725)	930. 6 (14.303)	920. 3 (13.632)
Fourth week	20	950. 2 (26.998)	931. 2 (14.722)	922. 1 (12.377)
Fifth week	20	953. 3 (28.507)	932. 8 (14.388)	923. 4 (12.445)
Sixth week	20	955. 3 (30.479)	934. 4 (13.936)	924. 5 (12.441)

(standard deviations are given within brackets).

**Table 4:** The mean weight changes in the three groups during the first week of observation (initially day to the sixth day).

Groups	Paired difference					*p*
	Mean difference	Std. error	95% confidence interval for the mean difference		*t*	
			Lower limit	Upper limit		
Group A	14.600	2.614	9.129	20.071	5.585	<0.001
Group B	17.350	2.141	12.868	21.832	8.102	<0.001
Group C	11.900	1.499	14.184	23.576	7.939	<0.001

**Table 5:** The mean weight changes in the three groups during the entire period of the observation (Initial day to the sixth week).

Groups	Paired difference					*p*
	Mean difference	Std. error	95% confidence interval for the mean difference		*t*	
			Lower limit	Upper limit		
Group A	26.150	3.565	18.688	33.612	5.585	<0.001
Group B	23.700	2.090	19.325	28.075	8.102	<0.001
Group C	18.850	2.229	14.184	23.516	7.939	<0.001
